# Impact of Immunoscore on the Management of Stage II Colon Cancer Patients: A Physician Survey

**DOI:** 10.3390/cancers13215467

**Published:** 2021-10-30

**Authors:** Anup Kasi, Efrat Dotan, Graham M. Poage, Aurelie Catteau, Dewi Vernerey, Manju George, Afsaneh Barzi

**Affiliations:** 1 Division of Medical Oncology, University of Kansas Medical Center, Westwood, KS 66205, USA; akasi@kumc.edu; 2 Fox Chase Cancer Center, Department of Medical Oncology, Philadelphia, PA 19111, USA; Efrat.Dotan@fccc.edu; 3 HalioDx Inc., Richmond, VA 23219, USA; graham.poage@haliodx.com; 4 HalioDx SAS, 13288 Marseille, France; Aurelie.Catteau@haliodx.com; 5 Methodology and Quality of Life in Oncology Unit, Besançon Hospital, 25000 Besançon, France; dvernerey@chu-besancon.fr; 6 Paltown Development Foundation, Crownsville, MD 21032, USA; manju@colontown.org; 7 City of Hope Comprehensive Cancer Center, Department of Medical Oncology & Therapeutics Research, Duarte, CA 91010, USA

**Keywords:** colonic neoplasms, oncologists, surveys and questionnaires, prognosis, tumor microenvironment

## Abstract

**Simple Summary:**

Selection of appropriate stage II colon cancer patients for adjuvant chemotherapy (AC) is controversial. A novel immune response classifier has previously been validated to refine patient selection, but its impact on oncologist treatment planning had yet to be described. In this survey, all but one oncologist altered clinical practice recommendations, and recommendations for AC prescriptions were reduced by half (among the Immunoscore-high cases (low recurrence risk)). This study revealed that the Immunoscore results could significantly decrease AC use in patients with stage II colon cancer who may not benefit from it, thereby reducing the administration of nonvalue care.

**Abstract:**

Background: Adjuvant chemotherapy use in stage II colon cancer is controversial. Current prognostic risk factors do not take the tumor immune microenvironment into account. Consideration of the Immunoscore, which measures the host immune response at the tumor site, may assist clinicians in reducing adjuvant chemotherapy use in patients who are unlikely to benefit from it. This study sought to determine the potential clinical utility of the Immunoscore, via its effect on medical oncologists’ recommendations for management of patients with stage II colon cancer. Methods: De-identified vignettes of 10 patients with stage II colon cancer were presented to 25 practicing medical oncologists. Each participant completed surveys indicating recommendations for adjuvant chemotherapy and surveillance strategies. An educational session was subsequently conducted, and the same patient profiles were re-presented but included immunoscore results. Participants were again asked to provide their recommendations. A participant was counted as influenced if their responses were altered after immunoscore test results were provided. Results: All but one participant (96%) altered a management recommendation for ≥1 case. For individual cases, a mean of 55% (range, 40–80%) of participants altered their recommendations for adjuvant chemotherapy and/or surveillance. For the immunoscore-high cases (low-risk of recurrence), recommendations for adjuvant chemotherapy use decreased from 60% to 31%. Conclusions: These results indicate a willingness by oncologists to integrate immunoscore information into clinical practice recommendations. Incorporation of immunoscore data resulted in the reduction of nonvalue care in the simulated population. Confirmation in prospective studies is planned.

## 1. Introduction

Each year in the United States, more than 100,000 patients are diagnosed with colon cancer [1], with approximately 25% having stage II disease. Although surgery alone cures 68–83% of these patients [2,3], they comprise of a heterogeneous population with variable outcomes, with 10–20% eventually developing metastatic disease [4]. While adjuvant chemotherapy (AC) with a fluoropyrimidine and oxaliplatin confers a clear survival advantage in most patients with stage III colon cancer [2], the use of AC in patients with stage II disease remains controversial [5,6,7]. Randomized trials [8], meta-analysis [9,10], and large retrospective studies [11,12] have failed to associate a robust survival advantage with AC in stage II colon cancer, though limited (e.g., 3–5%) improvement in survival has been reported [2,8]. Additionally, in contrast to the findings in stage III disease, the addition of oxaliplatin to fluorouracil (5FU) and leucovorin does not significantly benefit patients with stage II disease [13,14,15]. In a retrospective study of unselected SEER-Medicare patients with stage II disease, AC with fluoropyrimidines with or without oxaliplatin was not shown to provide a survival advantage [16].

Adjuvant therapy and follow-up recommendations require shared provider-patient decision-making, where individualized treatment plans incorporate patient, as well as provider, perceptions of potential risks and benefits. The decision to treat with AC in stage II colon cancer is largely based on recurrence risk factors. Tumor characteristics associated with increased risk of recurrence include pT4 stage; bowel perforation or obstruction; biomarkers for vascular invasion, lymphatic invasion, or perineural invasion; poor/undifferentiated grade; and inadequate lymph node sampling (fewer than 12 or 13) [2,17]. Neither American Society of Clinical Oncology nor European Society for Medical Oncology (ESMO) guidelines recommend the routine use of AC in medically fit patients with stage II colon cancer, but both recommend that physicians discuss it as an option for patients with high-risk disease, weighing the potential for benefits against known harms [2,17]. Adjuvant chemotherapy treatment duration also remains controversial. The International Duration Evaluation of Adjuvant Therapy (IDEA) collaboration trials of 12,835 stage III patients did not meet the primary endpoint of survival non-inferiority of de-escalated 3- versus 6-month treatment, although subgroup analyses suggested that lower risk patients (pT1-3N1) may be safely spared from a longer AC duration [18,19]. In a subset of the pooled studies, the analysis of patients with high-risk stage II disease who underwent 3 vs. 6 months of doublet AC (*n* = 3273) yielded similar results to the stage III studies, concluding that a better understanding of the relative contribution of the factors used to define high-risk disease in this setting is needed [20]. Toxicities, associated with AC in patients with colon cancer, range from quality-of-life impairment to financial toxicity and life-threatening harms [17]. Even single-agent fluoropyrimidine therapy, though lacking the long-term neurotoxicity seen with oxaliplatin-containing regimens [20], comes with risks (e.g., severe adverse reactions in patients with low or absent dihydropyrimidine dehydrogenase activity) [2]. Balancing risks vs. benefits is especially challenging in elderly patients, where life expectancy, comorbidities, and the fact that evidentiary randomized trials do not reflect a predominantly elderly population must be considered [5].

Significant improvements in clinical risk assessments that allow for a more accurate prognosis could better inform these discussions and the resulting treatment decisions. Immunoscore (IS) is a digital pathology diagnostic test that measures the host immune response at the tumor site through the automated quantitation of CD3+ and CD8+ tumor-infiltrating lymphocytes (TILs) at the invasive margin and tumor core, providing scores ranging from low (IS 0–1) to high (IS 2–4) [21,22]. A large international study validated the prognostic accuracy of IS in stages I-III colon cancer. In 1434 patients with stage II disease, high vs. low IS was associated with a significant lower risk of 5-year recurrence (hazard ratio, 0.49; 95% CI, 0.37–0.66; *p* < 0.0001) [23]. The impact of IS on prognosis in stage II disease was greater than that of all other prognostic factors combined, with a relative variable contribution to survival of 60% vs. 15.5% for poorly differentiated disease, the next most important factor [24]. Immunoscore identified a large subset of clinicopathologically high-risk stage II patients (70%) with a 5-year time to recurrence, similar to that of clinicopathologically low-risk patients [24] (89.1% vs. 87.4%, respectively). Among patients with T4N0 and IS-high colon cancers, the 5-year recurrence rate was low even without AC (12.5%) [25], while 59.2% of patients with IS-Low colon cancers recurred.

Based on available evidence, updated ESMO clinical guidelines recommend that IS be considered for use in conjunction with TNM staging for AC decision-making in patients with stage II, and some stage III, colon cancers [2]. The present study was initiated to gauge the potential clinical utility of IS and its effect on medical oncologists’ recommendations for management of patients with stage II colon cancer.

## 2. Materials and Methods

### 2.1. Survey Participants

This cross-sectional survey of U.S. medical oncologists treating gastrointestinal (GI) malignancies included physicians from a variety of practice settings (academic, community private practice, and community hospital health center). Medical oncologists who saw patients with GI cancers were identified using a database of public and commercially sourced information. Invitations to participate in the study were sent out and those agreeing to participate signed an agreement that included compensation for their time. Participants were accrued over a period of 3 months.

### 2.2. Cases and Assessments

Ten de-identified vignettes of patients diagnosed with stage II colon cancer for whom surgical samples had been submitted for IS testing were identified. No other inclusion or exclusion criteria were applied to the cases. Patient profiles were obtained from information submitted with the test samples, such as age, AJCC stage (lympho-vascular or perineural invasion), inadequate lymph node identification, poor differentiation, bowel obstruction, perforation, MSI/dMMR status, and comorbidities, where known. Prior to the live meeting, participants reviewed these vignettes, which included the clinicopathological case features, and provided their recommendation for AC or observation; if recommending AC, whether 5FU- or platinum-based chemotherapy should be used; if recommending AC, whether they would recommend a 3- or 6-month course; and whether they would recommend lenient (every 12 months for 5 years) or intensive (every 6 months for 2–3 years followed by every 12 months to complete 5 years) surveillance. Their responses were recorded via online survey forms. At an average interval of 1 week after completion of this survey, they attended a 45-min educational presentation by study authors on unmet needs in early colon cancer, the IS assay, and IS analytical performance and clinical validation studies in colon cancer. Immediately following the session, attendees were provided with the same 10 patient profiles, but this time with IS results (IS-high or IS-low) included in case summaries. Without any opportunity to view their prior responses, participants were once again directed to a survey with the same questions previously presented, regarding AC recommendation and surveillance intensity, and their responses were recorded.

### 2.3. Statistical Analysis

The primary study objective was to determine whether inclusion of IS results in decision-making would influence participants’ recommendations for the management of patients with stage II colon cancer for AC and surveillance. Secondary objectives included evaluating specific changes in recommendations for AC (i.e., observation, length of treatment (3 vs. 6 months), type of treatment (5FU- vs. oxaliplatin-based), and type of surveillance (lenient vs. intensive)) that IS data availability would make for each case and by each oncologist. 

An oncologist was counted as influenced by the IS assessment when there was ≥1 therapeutic modification (change in AC decision or surveillance level) after the IS test result (IS-high or IS-low) was provided. Decisions for individual cases, recorded before and after the IS educational presentation/data, were compared. It was hypothesized that a rate of medical oncologists influenced by IS assessment of 30% or more of participants (H1) would be considered effective, while 10% or less (H0) would not be effective, based on the following assumptions. According to the A’Hern design [26], with a one-sided alpha of 5% and 80% power, 25 physicians would need to be included to test the hypothesis. Should 6 or more participants be influenced (≥24%), per patient case, the IS result would be considered effective. To determine the associations between participant characteristics (sex, practice setting, and GI specialization) and recommendations for chemotherapy, before and after the IS information was provided, contingency tables were constructed, and two-sided Fisher’s exact tests were performed to determine significance for each of the 10 patient vignettes. An unsupervised, hierarchical clustering analysis was performed for participant recommendations of AC, among the 10 cases, to highlight different participant profiles.

## 3. Results

### 3.1. Participant and Case Characteristics

Twenty-five medical oncologists participated in the survey: 17 (68%) from academic medical centers, 4 (16%) in community private practice, and 4 (16%) from community hospital health centers. Fifteen participants (60%) listed their primary focus as GI. Nine participants (36%) were female. All participants recorded responses to every applicable survey question. Patient and disease characteristics for the 10 cases are summarized in Table S1. The median patient age was 63.5 years, 70% of the tumors were poorly differentiated, half had MSI-H/dMMR tumors, 3 patients (30%) had T4N0 tumors, 3 patients (30%) had lympho-vascular, perineural, or perivascular invasion, and 2 patients (20%) had reported comorbidities. No patients had macroscopic perforation or bowel obstructions.

### 3.2. Participant Changes in Recommendations for Adjuvant Chemotherapy Use and Surveillance

Of the 25 oncologists, 24 (96%) altered at least 1 AC (yes vs. no) or surveillance (lenient vs. intensive) recommendation for at least 1 case after IS educational training and data were made available, thereby exceeding the primary study endpoint of ≥30% to be considered significant.

In the pre-IS survey, as expected for current clinical practices [27], angiolymphatic invasion, T4 tumors, and poor differentiation status were the factors most commonly associated with recommendations for AC when considered independently (Figure S1). The total numbers of medical oncologist recommendations by case, pre- and post-IS, are shown in Table S2.

**Figure 1:**
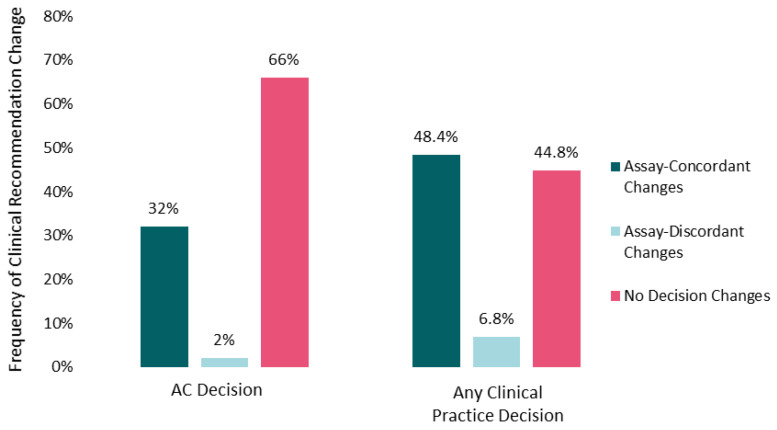
Impact of Immunoscore on clinical recommendations. Percentages of participants changing recommendations for adjuvant chemotherapy (yes vs. no on the left) or aggregated practice changes (any chemotherapy alteration or surveillance change on the right) after Immunoscore training and data were made available are shown. Assay-concordant changes represent participant recommendations that are congruent with the clinical implication of the Immunoscore data (e.g., no adjuvant chemotherapy for an Immunoscore-high patient tumor). Frequencies are averaged across the 10 case vignettes.

The mean number of cases for which oncologists altered practice recommendations post-IS was 5 (range, 0–10). More than half of participants changed recommendations for AC and/or surveillance intensity in 9 out of 10 cases. Changes to recommendations for AC (yes/no) were more frequent (34%) than changes to surveillance intensity (21%), and both types of changes were observed for immunoscore-high and immunoscore-low cases (Supplemental Table S2). Changes in chemotherapy (yes/no) recommendations (32% assay-concordant and 2% assay-discordant changes) contributed most to overall clinical practice modifications (48.4% assay-concordant and 6.8% assay-discordant changes), once assay information was taken into consideration (Figure 1). A mean of 55% of oncologists (range across the 10 cases, 40–80%) altered their recommendations for AC and/or surveillance across the 10 patient cases after reviewing the IS data.

**Figure 2:**
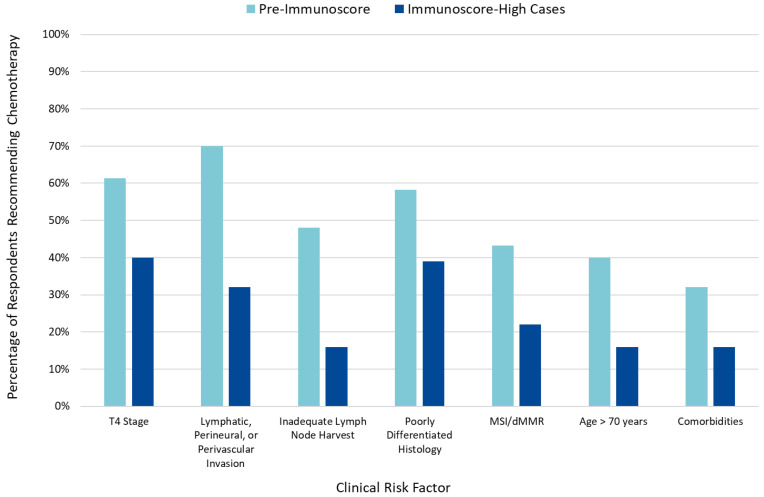
Impact of clinical risk factors pre- and post-Immunoscore on adjuvant chemotherapy recommendations.

When incorporated into clinical risk factor-based decision-making, an IS-high classification reduced the number of patients for whom AC was recommended (Figure 2), while an IS-low classification was associated with an increased number of patients for whom AC was recommended (Figure S2).

**Figure 3:**
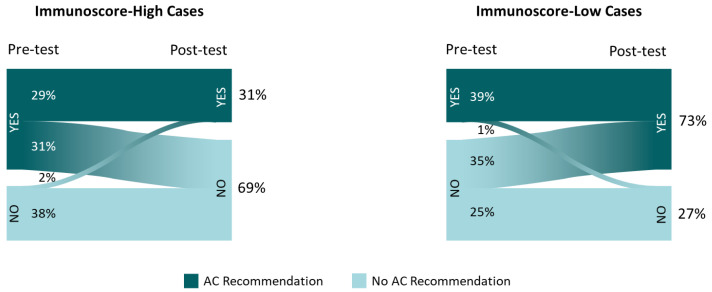
Adjuvant chemotherapy recommendations before and after Immunoscore availability in Immunoscore-high and -low cases. Inset frequencies represent total recommendations for adjuvant chemotherapy (vs. observation) among the 25 participants reviewing 10 cases.

Among the 7 cases with IS-high, the percentage of recommendations to treat with AC was reduced from 60% to 31%, following IS education/data review; in the 3 cases with IS-low, recommendations for AC increased from 39% to 73% (Figure 3). For 2% of the AC recommendation changes and 6.8% of any clinical practice changes, the type of change (escalation/de-escalation of treatment or surveillance) was discordant with the implied change in assay-suggested recurrence risk (higher/lower, respectively).

Hierarchical clustering revealed potential associations between participants’ sex/practice setting/GI-specialization and recommendations for AC (Figure S3). In 3/10 cases, the male sex was significantly associated with a higher frequency of baseline recommendations for AC (*p* < 0.05), but no association was observed between sex and a change in AC recommendation after participants received IS results. For 1 case, participant specialty was significantly associated with AC recommendations at baseline and after receiving IS results (*p* < 0.05), with physicians who were not GI-focused more frequently recommending AC at baseline and more frequently changing recommendations after IS results. Practice setting was not associated with baseline AC recommendations; however, in one case, participants in practice at community-associated sites more frequently changed AC recommendations than did participants associated with academic medical centers (*p* = 0.04).

### 3.3. Changes in Other Treatment Recommendations

**Figure 4:**
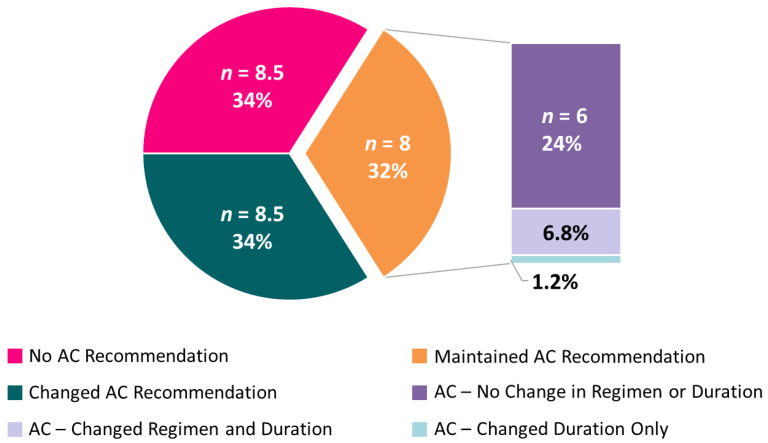
Average number (and mean percent) of participants across the 10 cases for adjuvant chemotherapy recommendations. Among those participants who recommended AC, both pre-test and post-test, nuanced recommendation changes are subset by regimen (single-agent vs. doublet) and duration (3 vs. 6 months).

For cases where AC was recommended both before and after IS status availability, IS data sometimes affected other recommendations. Upon receipt of IS results, 6.8% of participants changed their choice for the duration of recommended AC (3 vs. 6 months), and 1.2% changed their choice of singlet/combination chemotherapy (5FU- vs. oxaliplatin-based) (Figure 4).

**Figure 5:**
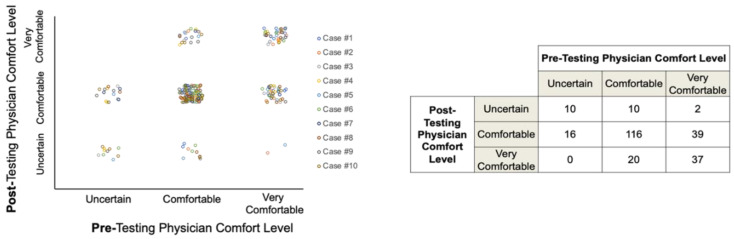
Participant comfort levels with treatment recommendations pre- and post-Immunoscore data availability.

The availability of IS status appeared to variably affect participant comfort levels with their management recommendations; however, most participants were comfortable or very comfortable with their treatment decisions both before and after IS data were revealed (Figure 5).

## 4. Discussion

Whether to offer AC is a difficult question that oncologists must confront when advising patients with stage II colon cancer [28]. The present study aimed to assess the effect of IS data on AC and surveillance recommendations by oncologists for patients with stage II colon cancer. Some 96% of the oncologists changed at least 1 management recommendation after reviewing IS data, suggesting that oncologists were willing to integrate a novel and potentially more informative prognostic marker in this complex setting. Thus, IS data availability had a significant effect on management recommendations. This endpoint, however, does not fully capture the potential clinical impact of IS data availability. To better quantitate this, we examined what percentage of oncologists altered their recommendations after reviewing IS data for each of the 10 cases. We found a change in management (AC or surveillance practices) was recommended by a majority of oncologists (mean, 55%) across the 10 cases they evaluated after IS data were provided. Immunoscore-high classification of patient tumors was associated with fewer recommendations for AC, while IS-low increased the likelihood that AC would be recommended. Immunoscore data availability, thus, appeared to be clinically meaningful.

The evolving role of adjuvant therapy in colorectal cancer and the development of new technologies for better risk stratification of patients adds to the complexity of decision-making in this population. We show that IS can improve the personalized management of patients with stage II disease by reducing nonvalue AC and improving surveillance practices.

The intent of AC is to treat residual micrometastatic disease to decrease risk of recurrence and achieve a cure. Approximately 80% of patients with stage II disease will survive for 5 years with surgery and no additional interventions [2,3]. Traditionally, prognostic risk factors, determined through retrospective analyses of large databases, were incorporated into the decision-making process for AC treatment. Although multiple studies have confirmed their prognostic value, their usefulness in guiding the decision on whether to recommend AC for patients with stage II colon cancer after surgery appears to be minimal, largely because most studies have associated little benefit with AC use in this population, regardless of risk status [15]. Nonetheless, current real-world practice employs AC, as seen in an analysis of Medicare beneficiaries with stage II colon cancer, of whom approximately 20% received AC within 6 months of surgery [29]. Interestingly, this study confirmed that when physicians are presented with information that suggests their patients are at a higher risk of recurrence than previously thought (i.e., IS-Low classification), treatment and surveillance recommendations were escalated, despite unproven benefits from additional interventions.

The risk-benefit decision, regarding the use of AC in the stage II setting, is more difficult in older adults who are most often seen in the clinic. Unbiased risk-benefit analysis, however, must consider both the direct and indirect costs during therapy, as well as the adverse effects associated with AC (specifically, the elevated risk for neuropathy associated with oxaliplatin) and financial toxicity for the patient and care givers. A recent evaluation of SEER data associated AC with approximately $26,500 higher costs in the first year, in comparison to observation among patients with stage II colon cancer [30].

The ability to better identify post-surgical patients with stage II colon cancer who are unlikely to progress, even without AC, could help guide treatment decisions and potentially reduce the burden of unnecessary intervention. Currently, accepted prognostic risk factors do not take into account the host immune response to the tumor, which encompasses the type, functional orientation, density, and location of adaptive immune cells within distinct tumor regions [31]. Immunoscore, which quantitates CD3+ and CD8+ TILs at the invasive margin and tumor core, was designed to account for this tumor immune contexture [31]. While tumor-informed prognostic factors will play an important role in prognostication of stage II patients, retrospective analyses have demonstrated that immune microenvironments assessed by IS are the stronger predictor of risk of recurrence [24]. Its prognostic accuracy in stages I-III colon cancer has been validated in multiple studies [23,32,33,34], and recent ESMO guidelines have recommended that it be considered, along with TNM staging, in decision-making for some patients with early-stage colon cancer [2].

Recent retrospective biomarker studies have examined the role of circulating tumor DNA (ctDNA) in the assessment of residual disease after surgery [35,36,37,38]. Though ctDNA positivity is prognostic and may help to select patients for AC, it has not yet been shown to predict survival benefit from AC. Keeping in mind the relatively low sensitivity of ctDNA detection at one time point (post-surgery), it is often recommended to serially monitor ctDNA for better sensitivity. Hence, ctDNA cannot inform decisions regarding use of AC, since detectable ctDNA is not universally shed by all tumors, detection may change with serial tests following surgery, and ctDNA may even persist after completion of AC [35]. Additional biomarkers are, therefore, needed for risk stratification and AC decision-making in this patient population. The complementary use of IS- and ctDNA-based assessment should be evaluated in future studies.

The role of immune-based classification for early-stage AC decision-making in the context of clinicopathologic markers is an area of active exploration. Microsatellite instability (MSI) occurs in a fraction of stage II colon cancers and identifies patients who have better outcomes than the overall population, while also predicting a lack of benefit from 5-FU-based therapies [39,40]. However, for the majority of patients, this marker is non-informative, due to the rarity of MSI. Cross-stratification analyses between immunoscore classification and MSI status have revealed that MSI status mis-assigns the recurrence risk for ~20% of patients, and the results from this survey study suggest that, when conflicting clinical markers are present (e.g., MSI-high and T4 stage) [23], immune classification is likely to change the expected treatment/surveillance decisions for these cases. This observation was replicated in the setting of other high-risk clinicopathologic factors, such as anigiolymphatic/perinural invasion, poorly differentiated histology, and inadequate lymph node examination. Arguably, the most important poor prognostic factor used by oncologists to select stage II patients for AC/high-frequency surveillance is T4 tumor stage and this factor was associated with pre-assay AC recommendations in this study. Interestingly, when the immunoscore classification indicated a low-risk for recurrence and high pre-existing immunity, treatment recommendations were altered by participants. This observation is supported by a recent analysis of the SITC validation study subset to the 208 T4N0, which revealed that 65% of these patients have a strong immune profile and patient survival times for these patients are consistent with low-risk cancers [25]. Tumor budding is a newer concept and is validated as an independent prognostic marker in patients with early-stage colon cancer. However, tumor budding was not predictive of benefit from adjuvant therapy in the SACURA study, a trial dedicated to stage II patients [41]. Correlative examination of the consensus Immunoscore and tumor budding are not available; however, histological analyses of tumor-infiltration lymphocytes (TILs) in large populations of early-stage colon cancers have been performed. These data clearly support the benefit of assessing both TILs and MMR/MSI status; TILs assessment provides greater prognostic relevance than does tumor budding [42,43].

## 5. Conclusions

Shared decision-making is the process of engaging patients in deciding about treatment plans. One limitation of the present analysis is that, owing to the nature of this study, the impact of a patient’s voice in the physician decision is unknown. Given the continued development of newer technologies for risk assessment, the complexity of decision-making for the oncologist is increasing and the influence of any added information, in this case, IS, may be less linear than predicted by this analysis. Finally, the number of participants and patient vignettes (representing a relatively narrow patient profile) evaluated in this study were limited, which could affect the generalizability of these results and there is a potential for biases in participant reporting, due to the nature of the study design (incentive and information anchoring).

Collectively, however, these results support the value of IS review in real-world clinical decision-making and its potential to decrease unnecessary use of AC and improve surveillance practices, thereby significantly reducing administration of nonvalue care. Future prospective studies, however, are needed to confirm these findings and define the role and value of IS in this setting.

## Data Availability

The data presented in this study are available in Supplemental Table S2.

## Supplementary Materials

The following are available online at www.mdpi.com/article/10.3390/cancers13215467/s1. Figure S1: Percentage of Participant Recommendations for Adjuvant Chemotherapy by Associated Patient Prognostic Risk Factors (Pre-test); Figure S2: Impact of Immunoscore-Low Classification on Adjuvant Chemotherapy Recommendations in Cases with Prognostic Risk Factors; Figure S3: Hierarchical Clustering of Participant Recommendations for Adjuvant Chemotherapy and Associated Demographic Factors; Table S1: Patient and Disease Profiles; Table S2: Changes in Medical Oncologist Recommendations Regarding Adjuvant Chemotherapy and Surveillance Intensity Post-Immunoscore by Oncologist and Case.
