# Supplementary material for: Impact of Immunoscore on the Management of Stage II Colon Cancer Patients: A Physician Survey
Source: Cancers (Basel). 2021 Oct 30;13(21):5467. doi: 10.3390/cancers13215467 (PMC8582441; doi:10.3390/cancers13215467)
Supplement: Supplementary file 1 [file cancers-13-05467-s001.zip › cancers-1420761-supplementary.pdf]

cancers MDPI

Supplementary Material: Impact of Immunoscore on the Management of Stage II Colon Cancer Patients: A Physician Survey

Anup Kasi, Efrat Dotan, Graham M. Poage, Aurelie Catteau, Dewi Vernerey, Manju George and Afsaneh Barzi

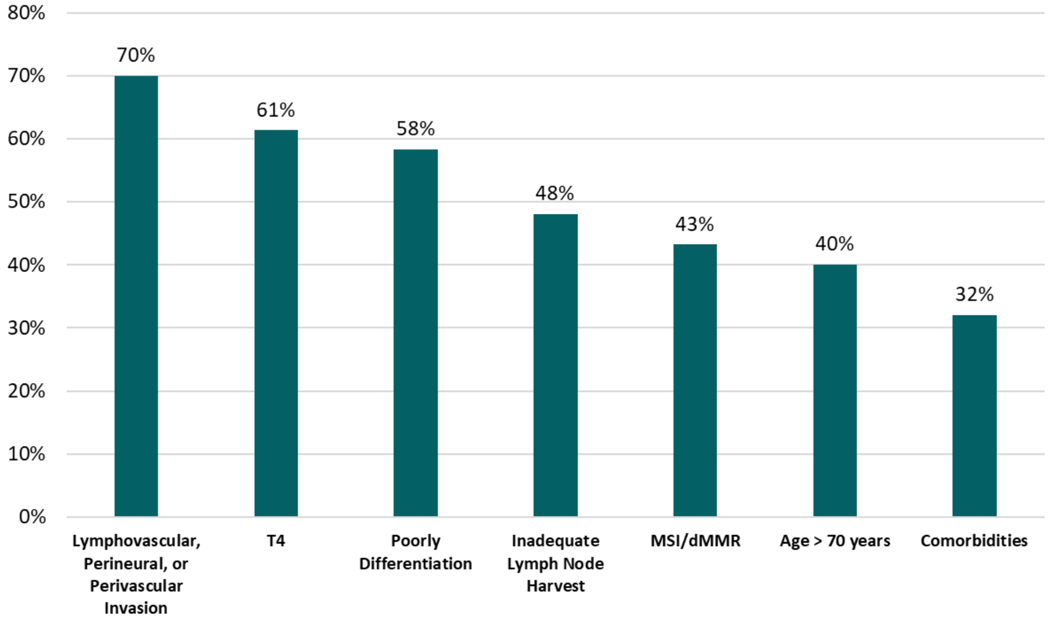

Figure S1. Percentage of Participant Recommendations for Adjuvant Chemotherapy by Associated Patient Prognostic Risk Factors (Pre-test).

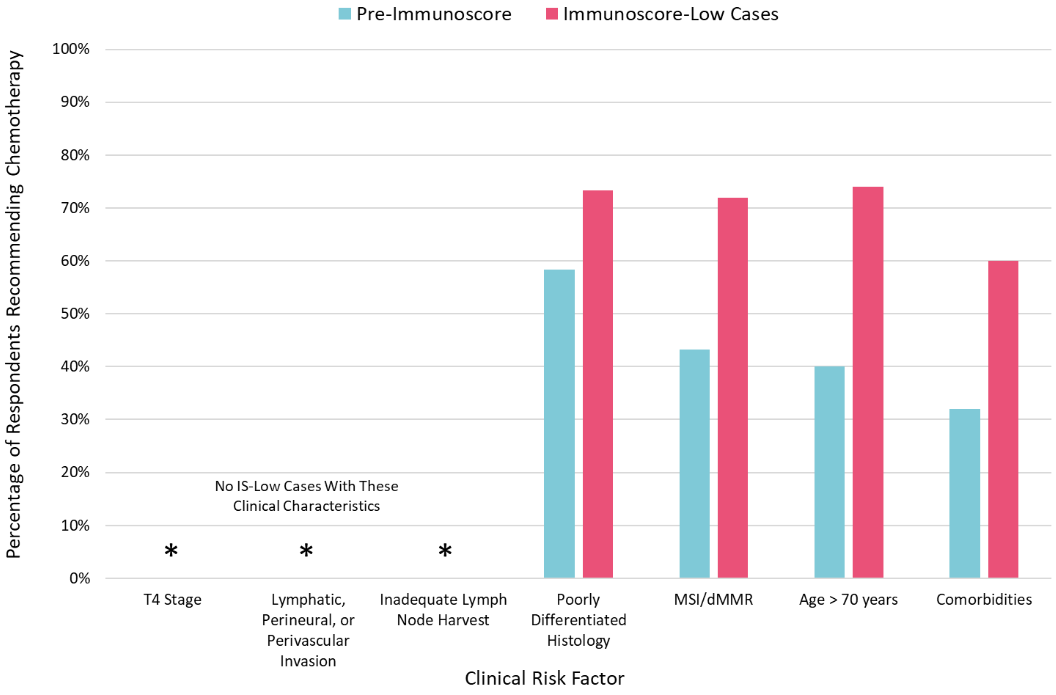

Figure S2. Impact of Immunoscore-Low Classification on Adjuvant Chemotherapy Recommendations in Cases with Prognostic Risk Factors. * No cases were represented with these clinicopathologic features.

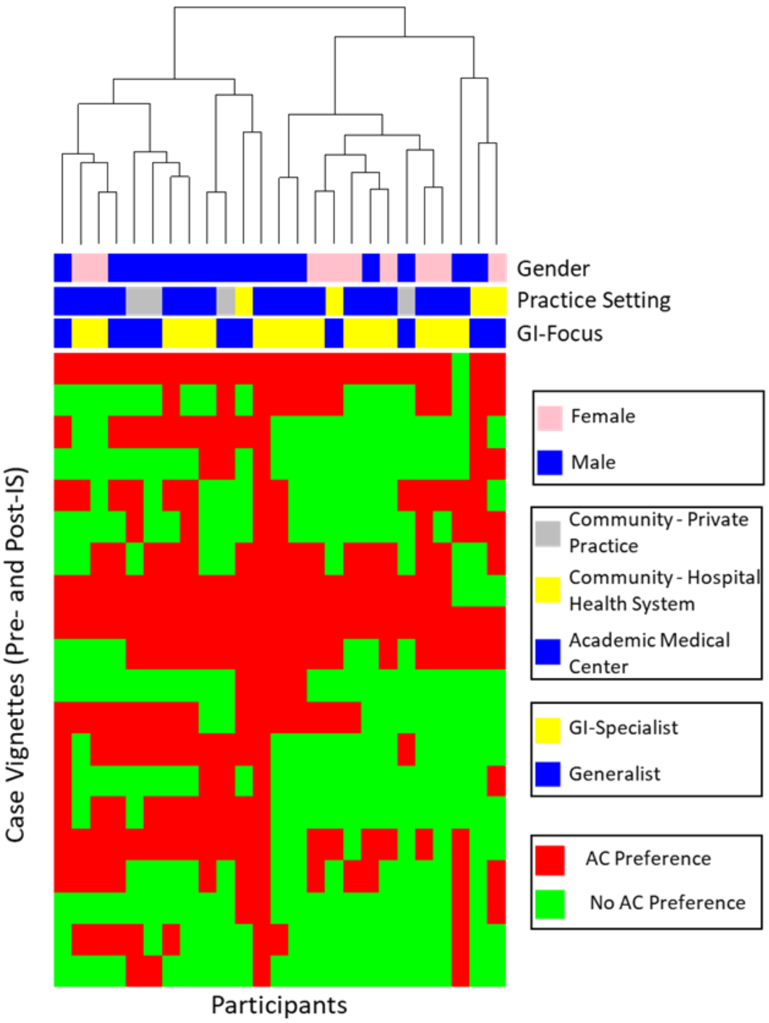

Figure S3. Hierarchical Clustering of Participant Recommendations for Adjuvant Chemotherapy and Associated Demographic Factors.

Table S1. Patient and Disease Profiles.

Characteristic	Value
Age	
Median (range), yr	63.5 (48-78)
>70 yr, *n* (%)	4 (40)
Comorbidities, *n* (%)	2 (20)
Tumor stage, *n* (%)	
T3	7 (70)
T4	3 (30)
Lymphatic, perineural, or perivascular invasion, *n* (%)	3 (30)
Inadequate lymph node harvest, *n* (%)	1 (10)
Bowel obstruction, *n* (%)	0
Perforation, *n* (%)	0
Poorly differentiated histology, *n* (%)	7 (70)
MSI-H/dMMR, *n* (%)	5 (50)
Immunoscore, *n* (%)	
Low	3 (30)
High	7 (70)

MSI/dMMR, microsatellite instability/deficient mismatch repair.

Table S2. Changes in Medical Oncologist Recommendations Regarding Adjuvant Chemotherapy and Surveillance Intensity Post-Immunoscore by Oncologist and Case.

Partici-pant	Case 1 (IS-High)	Case 2 (IS-High)	Case 3 (IS-High)	Case 4 (IS-Low)	Case 5 (IS-High)	Case 6 (IS-Low)	Case 7 (IS-High)	Case 8 (IS-Low)	Case 9 (IS-High)	Case 10 (IS-High)	Any Case
**1**	AC	AC	--	--	--	AC	AC	--	--	--	Yes
**2**	AC, S	--	--	S	AC, S	AC	AC	S	AC, S	AC	Yes
**3**	S	--	--	S	--	S	--	AC, S	--	--	Yes
**4**	AC, S	AC, S	AC, S	AC, S	AC, S	AC, S	S	S	AC, S	S	Yes
**5**	AC	--	AC, S	AC	AC	S	AC, S	S	--	S	Yes
**6**	AC, S	AC, S	S	--	S	AC, S	AC, S	--	S	AC, S	Yes
**7**	--	S	AC	S	S	S	--	S	--	S	Yes
**8**	AC	AC	--	AC	--	AC, S	AC	AC	--	--	Yes
**9**	--	--	--	--	--	--	--	--	--	--	No
**10**	--	--	--	AC	--	AC	--	AC	--	--	Yes
**11**	AC	--	--	S	--	S	--	AC, S	--	S	Yes
**12**	AC, S	S	AC, S	AC, S	AC	AC, S	S	AC, S	AC, S	AC, S	Yes
**13**	--	--	--	--	--	S	--	AC	S	--	Yes
**14**	AC, S	--	S	--	AC, S	--	--	AC, S	AC, S	S	Yes
**15**	--	AC	AC	--	--	AC	AC	--	--	AC	Yes
**16**	--	--	--	--	--	S	--	S	--	--	Yes
**17**	AC, S	AC, S	--	S	--	S	AC	--	--	--	Yes
**18**	S	S	S	--	S	--	--	--	--	AC	Yes
**19**	--	AC	AC	AC	--	S	AC	--	--	S	Yes
**20**	S	S	S	--	S	AC	S	AC	AC, S	S	Yes
**21**	S	S	S	--	S	AC	S	AC	--	--	Yes
**22**	--	--	--	AC	--	--	--	--	--	--	Yes
**23**	AC, S	AC, S	AC, S	--	AC, S	AC, S	AC	--	AC, S	AC, S	Yes
**24**	S	--	S	--	S	S	S	--	--	S	Yes
**25**	AC	--	--	--	AC	AC	--	--	AC	--	Yes
**Any Par-ticipant**	Yes	Yes	Yes	Yes	Yes	Yes	Yes	Yes	Yes	Yes	

AC, change in recommendation for adjuvant chemotherapy; S, change in recommendation for surveillance. --, no change in clinical recommendations
